# Effects of Land Use Type Transformation on the Structure and Diversity of Soil Bacterial Communities

**DOI:** 10.3390/life14020252

**Published:** 2024-02-13

**Authors:** Henian Hua, Xin Sui, Yanan Liu, Xu Liu, Qiuyang Chang, Ruiting Xu, Mengsha Li, Liqiang Mu

**Affiliations:** 1 Key Laboratory of Sustainable Forest Ecosystem Management-Ministry of Education, School of Forestry, Northeast Forestry University, Harbin 150040, China; hhn54553627@nefu.edu.cn (H.H.); lynm@nefu.edu.cn (Y.L.); liuxu19981209@nefu.edu.cn (X.L.); 1413085125@nefu.edu.cn (Q.C.); xrt@nefu.edu.cn (R.X.); 2 Engineering Research Center of Agricultural Microbiology Technology, Ministry of Education & Heilongjiang Provincial Key Laboratory of Ecological Restoration and Resource Utilization for Cold Region & Key Laboratory of Microbiology, College of Heilongjiang Province & School of Life Sciences, Heilongjiang University, Harbin 150080, China; xinsui_cool@126.com; 3 Institute of Nature and Ecology, Heilongjiang Academy of Sciences, Harbin 150040, China

**Keywords:** land use type, soil microorganisms, community structure, diversity

## Abstract

Soil microbiota are significantly influenced by their microenvironments. Therefore, to understand the impacts of various land use patterns on the diversity and composition of soil bacterial communities, this study focused on three typical land use types—NF (natural forest), AF (artificial forests), and FL (farmland)—in the Heilongjiang Central Station Black-billed Capercaillie National Nature Reserve, located in the southwestern part of Heihe City, Heilongjiang Province, China. Using high-throughput sequencing of the 16S rRNA gene, we examined the soil bacterial community structures in these different land use types and explored their correlation with soil environmental factors. The following were our main observations: (1) Significant variations in soil chemical properties among different land use patterns were observed. In artificial forests, total nitrogen (TN), alkali hydrolyzed nitrogen (AN), total phosphorus (TP), and available phosphorus (AP) were higher compared to farmland and significantly higher than those in natural forests. Furthermore, the organic carbon content (SOC) in natural forests was higher than in artificial forests and significantly higher than in farmland. (2) Comparative analysis using the Shannon and Simpson indices revealed that bacterial community diversity was higher in artificial forests than in natural forests, which was significantly higher than in farmland. (3) The effect of different land use types on soil bacterial community structure was not significant. The three land types were dominated by Proteobacteria, Acidobacteria, and Actinobacteria. Proteobacteria exhibited a higher relative abundance in farmland and artificial forests compared to natural forests, whereas Actinobacteria exhibited the lowest relative abundance in natural forests. (4) Redundancy analysis (RDA) revealed that SOC, TN, AN, and AP were key environmental factors influencing the microbial communities of soil. Collectively, our findings demonstrated that land use practices can significantly alter soil nutrient levels, thereby influencing the structure of bacterial communities.

## 1. Introduction

Changes in land use have a profound impact on terrestrial ecosystems and biogeochemical cycles, thereby altering soil properties and fertility [1]. These alterations, in turn, influence the diversity and composition of soil microbial communities [2]. Soil microorganisms are a crucial component of soil ecosystems and are intricately linked to plant growth and soil biogeochemical cycling [3]. Moreover, the composition and structure of soil microbial communities significantly shape soil structure and functionality [4,5]. Therefore, the impact of different land use modes on the structure and functionality of soil ecosystems can be predicted by observing structural changes in soil microorganisms [6].

Different land use practices induce alterations in vegetation coverage and soil physicochemical properties. These changes are closely linked to the environmental heterogeneity of soil and represent pivotal factors influencing the structure of the microbial communities of soil [7,8]. For example, ref. [9] observed significant variations in soil bacterial diversity and community distribution across different land use types in the Chongqing Yangtze River Basin. Furthermore, the authors noted a consistent relationship between changes in bacterial diversity and alterations in soil physicochemical properties. Additionally, α-diversity is commonly utilized as a basis to analyze soil microbial diversity. Li Jinbiao et al. [10] found that alterations in land use types lead to changes in soil chemical properties, significantly impacting the composition and diversity of soil bacterial communities. The chemical properties most affected by changes in land use types were soil organic carbon content, followed by total phosphorus, total nitrogen, and total potassium. Moreover, Wu Yining et al. analyzed microbial community composition and functionality in marsh wetlands, meadow wetlands, forests, and farmlands and reported that soil bacterial abundance in forested areas significantly surpassed that of other land use patterns. Additionally, the authors noted that total nitrogen significantly influenced both the diversity and abundance of soil microbial communities [11]. Multiple studies have characterized the effects of anthropogenic activities on the diversity and composition of soil microbial communities, including agriculture, urban development, industry, and pesticide use [12,13]. Nevertheless, the complex environmental factors and management practices involved in different land use practices may have synergistic effects on the microbial composition of soil, and therefore more comprehensive studies are needed to understand this complex relationship. Understanding the effects of land use practices on soil microorganisms can thus provide a theoretical and practical basis for the management and conservation of soil ecosystems.

Heilongjiang Province boasts a rich agricultural history and fertile land, making it one of China’s foremost centers for commodity grain production. The soil’s physicochemical properties and fertility in this region are of paramount importance for the advancement of agriculture [14]. The Heilongjiang Central Station Black-billed Capercaillie National Nature Reserve is located in the transitional zone between the Greater and Lesser Khingan Mountains. The primary dominant community within the reserve comprises the Dahurian larch forests. Moreover, the Central Station stands as one of the oldest regions in Northeast China in terms of historical development and utilization. However, it is also considered an ecologically fragile environment. Due to population growth, this region has resorted to deforestation and land clearance to enhance agricultural yields and address food security concerns. Human intervention has resulted in the degradation of primary forests, leading to the emergence of extensive secondary vegetation, such as birch and Mongolian oak forests. These alterations in forest types not only impact ecosystem functionalities but also exert a notable influence on the soil ecosystem [15]. Furthermore, the conversion of natural forests into agricultural fields has led to the loss of natural vegetative cover, leaving the soil surface exposed and causing severe detriment to the soil ecosystem.

Based on the aforementioned background, our study sought to comparatively analyze the bacterial community composition and diversity across three distinct land use patterns within the Central Station area (natural forest, plantation forest, farmland) using a high-throughput sequencing approach. Particularly, this study aimed to establish a scientific basis for the preservation of soil fertility, nurturing practices, and the conservation of soil microbial diversity to enable ecological reconstruction and sustainable land resource utilization in the study area.

## 2. Materials and Methods

### 2.1. Study Site

The study site was situated in the Heilongjiang Central Station Black-billed Cap-ercaillie National Nature Reserve, located in the southwestern part of Heihe City, Heilongjiang Province. This site lies within the transitional zone between the southwestern foothills of the Lesser Khingan Mountains and the Songnen Plain, encompassing a total area of 988.6 km^2^. The region is located within the temperate continental monsoon climate zone, characterized by prolonged and harsh winters as well as brief and cool summers. The average annual temperature is −0.5 °C, with a frost-free period lasting 121 days. The annual average rainfall is 476.33 mm, and the average relative humidity is 69.2%. The nature reserve features a typical alpine forest ecosystem, encompassing forests, shrubs, wetlands, and meadows. The forests include coniferous forests, coniferous and broad-leaved mixed forests, and broad-leaved forests, boasting a high forest coverage of 82.4% [14]. The main plant species in the study area include *Larix gmelinii* larch, *Betula platyphyllabirch*, black birch, *Betula dahurica*, *Quercus mongolica*, Mongolian oak, Amur linden (*Tilia amurensis*), diamond willow (*Chosenia arbutifolia*), aspen (*Populus davidiana*), bird cherry (*Prunus padus*), *Salix raddeana*, and alder trees (*Alnus mandshurica*).

### 2.2. Sample Collection

In June 2022, three typical land use types: plantation *Larix gmelinii* forest, natural forest (mixed forests of *Larix gmelinii* forest, *Betula platyphylla* forest, and *Betula dahurica* forest), and agricultural land (soybean cultivation) were selected in this nature reserve. Each land use type included three independent replicates, and each replicate also included three standard quadrats, so a total of nine standard quadrats were established. Each quadrat set is 50 × 50 m. Soil samples were collected at a depth of 0–20 cm using a soil shovel with a five-point sampling method, which were mixed as one soil sample. After collection, the soil samples were first cleaned of plant debris and then passed through a 2 mm sieve. The processed soil samples were then sealed in ziplock bags and stored at −20 °C in the laboratory for subsequent analysis.

### 2.3. Analysis of Soil Physicochemical Properties

Soil total nitrogen was determined using the Kjeldahl method, whereas available nitrogen was assessed using the alkaline hydrolysis diffusion method. Soil total phosphorus was determined via the sulfuric acid-perchloric acid digestion method followed by the molybdenum-antimony colorimetric technique, while available phosphorus content was quantified using the bicarbonate method. The total potassium content of the soil was analyzed using the sodium hydroxide fusion-flame photometer method. Available potassium was quantified using ammonium acetate extraction, followed by flame photometry. Soil organic carbon content was measured with additional heating using an oil bath.

### 2.4. Sample DNA Extraction

Genomic DNA was extracted using the E.Z.N.A.^®^ Soil Kit (Omega Bio-tek, Norcross, GA, USA). The quantity and integrity of the extracted DNA were then preliminarily examined using 1% agarose gel electrophoresis. Afterward, the DNA was precisely quantified using the QuantiFluor™—ST Blue Fluorescence Quantitation System (Promega Corporation, Madison, WI, USA). Finally, the samples were proportionally mixed according to the sequencing requirements for subsequent experiments.

### 2.5. 16RsRNA Sequence Amplification and Library Construction

To prevent excessive sequence length for sequencing while maintaining amplification heterogeneity, the primer set 515F/907R was selected for amplifying the bacterial 16S rDNA fragment from total DNA samples. Polymerase chain reaction (PCR) amplifications were conducted using the following gene-specific primers: 515F: 5′-GTGCCGCCAGCMGCCGG-3′, 907R: 5′-CCGTCAATTCMTTTRAGTTT-3′. PCR reactions were prepared in triplicate with a 20 μL reaction volume, and the DNA template concentration was 10 ng. To ensure accurate and reliable data, a lower cycle number was employed for amplification, aiming for uniform cycle numbers across all samples. After preliminary experimentation, 25 cycles were determined to be optimal. The PCR protocol consisted of an initial denaturation step at 95 °C for 2 min, followed by 25 cycles of denaturation at 90 °C for 30 s, annealing at 55 °C for 30 s, extension at 72 °C for 30 s, and a final extension at 72 °C for 5 min. The PCR products from the same sample were pooled and analyzed using 2% agarose gel electrophoresis. Gel extraction of PCR products was conducted using the AxyPrepDNA Gel Recovery Kit (Axygen Biosciences, Union City, CA, USA), followed by elution in Tris-HCl buffer and subsequent analysis via 2% agarose gel electrophoresis. For accurate quantification, the PCR products were quantified using the QuantiFluor™—ST Blue Fluorescence Quantitation System (Promega Corporation), based on the preliminary results obtained from gel electrophoresis. Subsequently, the samples were proportionally mixed according to the sequencing requirements for each sample. The mixed samples were subjected to high-throughput sequencing using the Illumina HiSeq 2500 platform (Shanghai BIOZERON Biotech., Shanghai, China). This sequencing strategy aimed to balance sequence length for effective sequencing while ensuring adequate representation of genetic diversity across the samples. All the sequences were deposited in the NCBI, and the serial number was PRJNA1059507.

### 2.6. Sequencing Data Processing and Analysis

The data extracted based on index sequences were stored in fastq format. Quality control filtering was performed on reads using barcode information to distinguish between different samples. After filtering, paired-end (PE) reads were analyzed to identify overlapping regions and subsequently merged into a single sequence. High-quality sequences were obtained for each sample by splitting based on barcode and primer sequences. Sequence directionality was corrected based on the orientation of the forward and reverse barcodes and primers, while chimeras were removed during this process. A quality control filter was applied to trim bases with a quality value below 20 at the end of the reads. Additionally, a sliding window of 10 base pairs was implemented to identify regions with an average quality value lower than 20, and bases beyond this window were trimmed. Reads shorter than 50 base pairs after quality control were filtered out. The PE reads were consolidated into a single sequence with a minimum overlap length of 10 base pairs. The maximum allowable mismatch rate in the overlapping region of merged sequences was set to 0.2, and sequences not meeting this criterion were removed. Samples were demultiplexed based on barcode and primer regions, with barcode mismatch set to zero and a maximum primer mismatch of 2. A combined de novo and reference approach using Usearch (V10) software and the Gold database was employed to remove chimeric sequences. This rigorous process ensured the generation of high-quality, non-chimeric sequences suitable for subsequent analysis.

For soil alpha diversity analysis, Chao1, Shannon, Simpson, and ACE indices in soil samples were determined using Mothur (version 1.35.1). This analysis was conducted using the Uclust algorithm (version 1.2.22q) to categorize species based on representative operational taxonomic unit (OTU) sequences at a 97% similarity threshold. Analysis of species composition and disparities within each soil sample was performed at both the phylum and genus levels. Additionally, redundancy analysis across different samples was conducted using the Canoco 5.0 software. Basic data analysis was conducted using Excel 2020, whereas the SPSS 26.0 software was employed for statistical analysis. Origin 2019 and the R programming language (3.6.3) were used for data visualization. This comprehensive array of tools and software enabled a thorough exploration and understanding of soil microbial communities and their diversity.

## 3. Results

### 3.1. Physicochemical Properties of the Soil

The physicochemical properties of soil varied significantly depending on land use (Table 1). Specifically, TN and AN reached their highest levels in industrial forests at 4.17 g/kg and 156.33 g/kg, respectively, whereas natural forests exhibited the lowest values of 1.67 g/kg for TN and 85.17 g/kg for AN. SOC reached its peak concentration in natural forests at 71.47 g/kg, contrasting with its lowest value in agricultural land, measuring 43.64 g/kg. TP content was 0.59 g/kg for farmland and 0.26 g/kg for natural forest. Lastly, TK exhibited the highest (9.76 g/kg) and lowest (6.25 g/kg) concentrations in natural forests.

### 3.2. Microbial α-Diversity Analysis

Significant differences were observed among the soil bacterial α-diversity of different land uses (Table 2). The Chao1 index reached its peak at 4475.56 in agricultural land, whereas the lowest value (3775.49) was recorded in natural forest. Moreover, the highest Shannon’s diversity index of 10.44 was observed in natural forests, whereas the lowest value (9.55) was detected in agricultural land. Similarly, Simpson’s index exhibited the greatest diversity at 0.56 in natural forest, contrasting with its lowest value (0.027) in agricultural land.

### 3.3. Structural Composition of Microbial Communities

**Figure A1:**
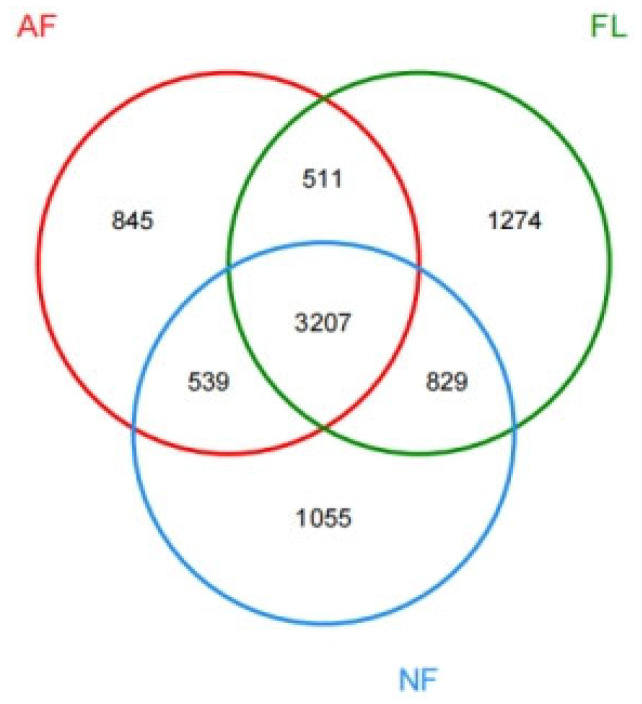
Venn diagram of the OTU distribution.

The results of our high-throughput sequencing analyses yielded a total of 334,356 valid sequences from all of the examined soil samples, with an average length of 363.88 bp. Out of these sequences, 8260 sequences were categorized as OTUs and further classified into 41 phyla, 102 orders, 232 orders, 296 families, 523 genera, and 601 species (Figure A1). The number of OTUs varied among the different land use types: 5821 species in agricultural land, 5630 species in natural forests, and 5102 species in planted forests. There were a total of 3207 OTUs in the three sampling plots, with agricultural land, natural forests, and planted forests encompassing 1274, 1055, and 845 OTUs, respectively (Table A1).

**Figure 1:**
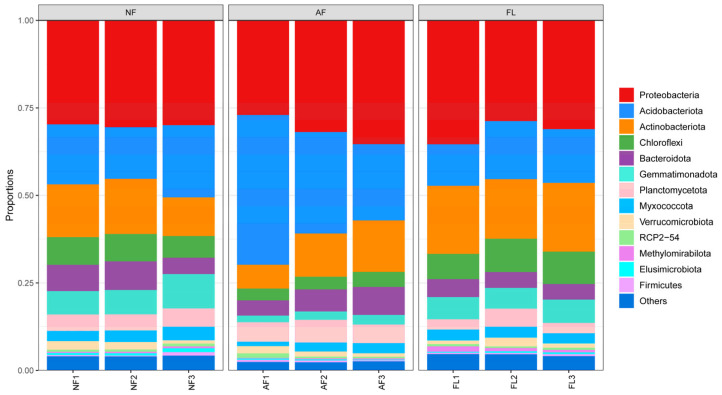
Bacterial community composition in different soil samples at the phylum level.

As outlined in Table 3, significant differences were observed in the levels of bacterial phyla under different land uses, with Myxococcota, Acidobacteriota, Actinobacteriota, Chloroflexi, Gemmatimonadota, and Planctomycetota showing significant differences. In contrast, Bacteroidia, Proteobacteria, and Verrucomicrobiota were not significantly different (Table A2). The Acidobacteria phylum exhibited the highest abundance (31.2%) in natural forests and the lowest (14.6%) in agricultural land. The Actinobacteria phylum displayed the highest abundance (18.7%) in agricultural land and the lowest (11.8%) in natural forests, whereas the Gemmatimonadota phylum was the highest at 8.6% in agricultural land and the lowest at 3.8% in artificial forests (Figure 1).

**Figure 2:**
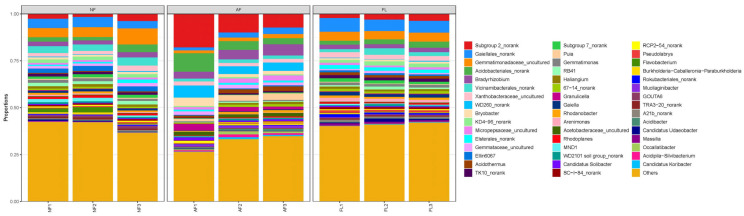
Bacterial community composition in different soil samples at the genus level.

At the genus level (Figure 2), bacterial taxa with a relative abundance greater than 5% included *Sub-group 2_norank* (11.69%), *Gaiellales_norank* (6.6%), *Gemmatimonadaceae_uncultured* (6.21%), *Acidobacteriales_norank* (5.94%), and *Bradyrhizobium* (5.13%). Bacterial taxa with a relative abundance of less than 1% accounted for more than 30% of the three land use types.

### 3.4. Correlation between Soil Physical and Chemical Properties and the Relative Abundance of Microbial Communities

**Figure 3:**
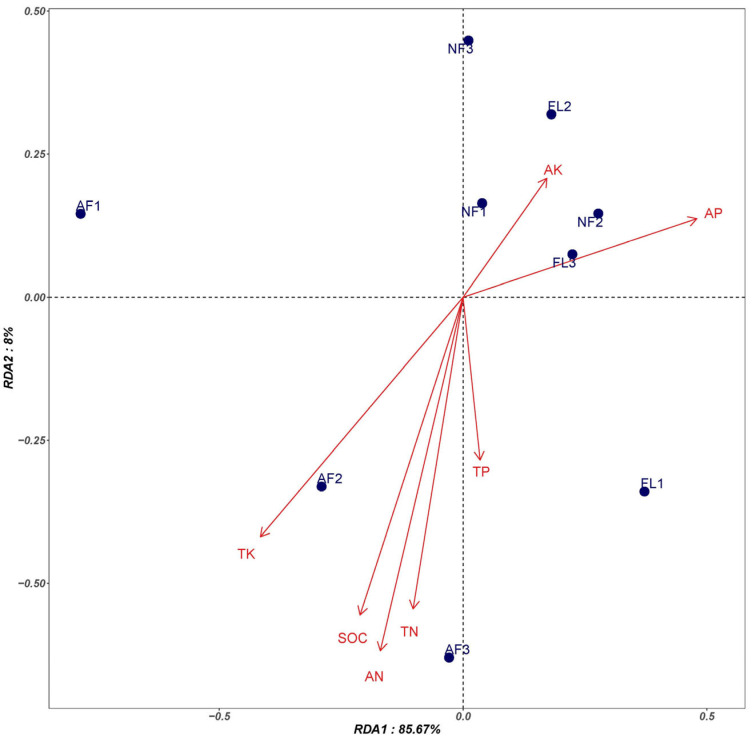
RDA of microbial community composition and soil physicochemical properties.

Redundancy analysis (RDA) of the soil biological communities of the three land use patterns at the genus level was conducted to preliminarily analyze the effects of soil physicochemical factors on soil microbial communities (Figure 3). The RDA results indicated that the two axes explained a total of 93.67% of the community changes, with the vertical axis explaining 8% of the community changes and the horizontal axis explaining 85.67% of the community changes. The composition of the bacterial community of natural forests was primarily influenced by AP and AK, whereas that of artificial forests was influenced by TK, AN, TN, and SOC, and the bacterial community composition of farmland soil was mainly influenced by TP, AP, and AK.

Spearman correlation analysis was conducted to reveal the relationship between the microbial community composition and the physical and chemical properties of the three soil types depending on land use (Table 3). The results revealed that the Chao1 index and Shannon index were not significantly correlated with physical and chemical properties. However, significant correlations existed between the dominant bacterial groups (Mucor, Actinobacteria, and Bacillus) and soil physicochemical properties. In contrast, the dominant bacterial phyla such as Aspergillus and Acidobacteria, did not exhibit significant correlations with any of the examined physicochemical properties. Specifically, the genus Mucor exhibited a significant negative correlation with total potassium content (*p* < 0.05), whereas the phylum Actinobacteria exhibited a significant positive correlation with quick-acting phosphorus content (*p* < 0.05). The phylum Bacillus displayed a significant negative correlation with quick-acting nitrogen content (*p* < 0.05), a highly significant negative correlation with total potassium content (*p* < 0.01), and a significant negative correlation with soil organic carbon content (*p* < 0.05).

## 4. Discussion

Soil microorganisms are crucial components of ecosystem dynamics and play a pivotal role in plant and soil nutrient cycling metabolism [16,17], with bacteria being among the most diverse and numerous microorganisms in soil [18]. Their multifaceted impact extends to plants, animals, and soils, influencing soil nutrient cycling, fertility maintenance, and enhancement, as well as plant growth and development [19].

This study revealed Proteobacteria, Acidobacteria, and Actinobacteria as dominant bacterial phyla in soils of different land use types. However, land use significantly altered the relative abundance of soil bacterial phyla, which was consistent with the results of Zou, Xiaoxiao and Cai, Guanxia [20,21,22,23]. Moreover, we summarized other studies in different regions and found that the dominant soil bacterial phyla were all the same in different land use types, but the structure and diversity of soil microbial communities changed significantly (Table A3). In this study, we observed substantial changes in the relative abundance of dominant phyla, with the highest relative abundance of Actinobacteria reaching 18.7% in agricultural land, whereas the lowest level of 14% was observed in natural forests. This variation may be attributed to manual straw crushing practices in farmland, leading to the highest relative abundance of Actinobacteria, which accelerates apoplastic material decomposition [24]. It may also be related to the potential ecological properties of actinomycetes, where bacteria of the phylum Actinomycetes play a key role in organic matter decomposition, facilitating the decomposition and cycling of organic matter. The highest relative abundance of Acidobacteria was identified in artificial forests at 31.2%, contrasting with the lowest in agricultural land at 14.6%. Although previous studies have suggested that Acidobacteria abundance is higher in nutrient-poor environments [25,26,27], the present findings align with Junjie Liu et al.’s observations in black soil, indicating a unique distribution pattern and a correlation with soil nutrient content [28]. It may also be related to the life habits of Acidobacteria, which are usually abundant in more acidic soils and are adapted to acidic environments. The highest abundance of Proteobacteria was observed in agricultural land at 31.8% and the lowest in natural forest at 30%. This may be linked to Proteobacteria preference for eutrophically rich soils [29], which is consistent with the relatively high nutrient content in agricultural land (nitrogen, phosphorus, and potassium). The adaptability of Proteobacteria, often parthenogenetic or aerobic bacteria, could also contribute to their higher abundance [30]. Additionally, artificial fertilization in farmland may have influenced the soil structure, enhancing the suitability of green leafy plants, known for their stress tolerance, in nutrient-poor environments [31]. It is also possible that this may be related to the cultivation of soybean at the site, which is a nitrogen-fixing plant, and that some members of the phylum Ascomycota are involved in nitrogen fixation and nitrification-reduction processes, which have an important influence on the nitrogen cycle in the soil. However, the slightly higher relative abundance of the Gemmatimonadota phylum in agricultural land may be related to organochlorine pesticide use, as its abundance has been shown to increase with the application of organochlorine [32].

From Table 3, Actinobacteriota, Myxococcota, and Gemmatinnonadota were more strongly correlated with soil chemical properties. Among them, Actinobacteriota showed some degree of positive correlation on AK, AN, TN, TP, TK, AP, and SOC, especially on AP and SOC. This may be due to the fact that some members of Actinobacteriota may have the potential to decompose organic phosphorus. Organic phosphorus is an important pool of phosphorus in the soil, and its degradation helps to provide plant-available inorganic phosphorus. Thus, the positive role of Actinobacteriota may involve the production of organic phosphorus-degrading enzymes that promote efficient phosphorus utilization in the soil. On the other hand Actinobacteriota are usually known for decomposing organic matter. They may be involved in the decomposition of complex organic matter in the soil, producing secondary metabolites, and contributing to the soil carbon cycle. The positive correlation in terms of SOC may be related to the involvement of Actinobacteriota in organic matter degradation. They may decompose residues and organic matter and promote the decomposition and mineralization of organic matter in the soil. Myxococcota may be more sensitive to the utilization of fast-acting potassium. This may involve a decrease in the relative abundance of Myxococcota in areas enriched in fast-acting potassium, as their metabolic activities are affected by fast-acting potassium concentrations. The negative correlation of Myxococcota may be related to their specific functions in the nitrogen, phosphorus, and carbon cycles. Possible mechanisms include their utilization of certain nutrients in the soil or their sensitivity to specific environmental conditions. Gemmatinnonadota showed negative correlations for a wide range of soil nutrients (AK, AN, TN, TP, TK, AP), which may be due to its high sensitivity relative to the concentrations of these nutrients. It is possible that its metabolic activities are directly affected by changes in the concentrations of these nutrients. The negative correlation with TK and SOC may indicate that Gemmatinnonadota is highly sensitive to organic carbon levels in the soil. This group of bacteria may increase in relative abundance in environments with relatively low SOC in response to decreasing organic carbon levels.

At the genus level, a total of 523 microbial genera were detected in natural forests, artificial forests, and farmland under different land use modes over the long term, and the distribution patterns of these microbial genera were notably distinct. Subgroup 2_norank exhibited the highest relative abundance in plantation forest soils and belonged to the Acidobacteria phylum. This observation may be attributed to the predominance of eutrophic Acidobacteria in natural forests, where nutrient content is relatively high. It could also be linked to the unique distribution pattern of Acidobacteria in black soils [28], consistent with our study’s findings. The relative abundance of Gaiellales_norank was higher in farmland than in natural and artificial forests, potentially connected to farmland tillage. Aerobic bacterium characteristics of Gaiellales_norank may be favored by crop tillage, increasing soil oxygenation [33].

Differences in soil utilization not only impact variations in microbial community structure but also influence the diversity of soil microbial communities [34]. In this study, significant differences in soil microbial community diversity were observed among different land use modes. The bacterial diversity of natural forests was higher than in other land use modes, consistent with the results of Jiao et al. [35]. The higher bacterial diversity in natural forests may be attributed to long-term management practices such as artificial nurturing and inter-logging, which influence the soil microbial community and promote or inhibit the growth of specific microorganisms, thus enhancing bacterial diversity [30]. Land use practices have substantial effects on soil chemical properties (e.g., TN, AN, SOC, TK, etc.), as well as on soil microbial diversity. Our RDA results revealed that soil SOC, TN, AN, and TK had a significant impact on soil bacterial diversity, aligning with previous studies indicating the strong influence of soil nutrients on the diversity and abundance of soil microbial communities [36]. Here, we observed that the lowest bacterial diversity index in farmland may be attributed to the lack of nutrients and organic matter in the soil due to prolonged cultivation, even with artificial fertilization. Additionally, factors such as the absence of ground cover, prolonged exposure of the soil surface to sunlight, and soil dryness may hinder the survival and reproduction of bacteria. The SOC content may also play a role, as studies suggest that the conversion of forests into farmland leads to a 25% to 50% decrease in SOC content in the soil [37]. Wang G et al. [38] found that lower SOC content may reduce the number of antagonistic bacteria in the soil, subsequently diminishing soil bacterial diversity. The results of this study suggest that changes in nutrient elements and chemical properties in soils of different land use types may directly affect the ecological niche and metabolism of microorganisms. Also, it may be related to vegetation types, which provide different root secretions and organic matter, which directly affect the type and number of microorganisms in the soil.

Related studies have indicated that soil microbial abundance increases after converting native vegetation to agricultural land [39], aligning with the findings of this study. The highest bacterial abundance was observed in agricultural land, possibly linked to anthropogenic disturbance. Lammel et al. noted a substantial increase in soil bacterial abundance from primary rainforests and savannahs to agricultural land in the southern part of the Amazon due to sustained anthropogenic disturbance [40]. Frequent anthropogenic disturbances may stimulate the enrichment of bacterial OTUs [41]. Natural forests exhibited relatively lower bacterial abundance, possibly due to the variable responses of different bacterial communities to changes in soil nutrient levels. Changes in the soil microenvironment, including nutrients, can also influence microbial diversity [42]. In this study, Spearman analysis indicated that SOC and TN had a lesser effect on soil diversity, contrary to previous studies suggesting a greater impact of soil nutrients on the diversity and abundance of soil microbial communities [43]. Seasonal factors must also be considered, as highlighted in the study by Ji Chuning et al., where pH and moisture content significantly affected the diversity of soil fungal communities during fungal community succession in reclaimed soils in a mining area [44]. Various factors, including seasonal variations, land use, and management practices, significantly impact bacterial abundance and diversity [45,46]. Sampling was conducted during the summer season, when plants naturally grow and differences in vegetation can cause changes in soil physicochemical properties, affecting soil microorganisms. Agricultural management practices and variations in climatic conditions during the summer may alter soil properties, subsequently influencing soil microorganisms. Although this study provides clear evidence that land use type significantly affects soil microbial community composition, there are still several uncertainties and limitations that must be addressed. The response of the microbial community to land use changes is time-dependent. However, this study only examined short-term responses. To better understand the impact of different land use types on the structure and diversity of soil microbial communities and provide a reliable basis, long-term and continuous monitoring across various land use types is essential.

## 5. Conclusions

Our findings demonstrated significant differences in soil bacterial diversity among different land use modes in the Black-billed Grouse Nature Reserve at the Central Station of Heilongjiang. Soil bacterial diversity in artificial forests was significantly higher than that in farmland, indicating a notable variation in microbial richness. The nutrient content of soil in artificial forests and farmland exhibited a significant increase compared to that in natural forests. Additionally, microbial abundance in farmland was significantly higher than that in artificial forests. Meanwhile, the dominant microbial taxa in natural forests, plantation forests, and farmlands were essentially the same, but their community structures differed. The primary environmental drivers for these variations might include TN, AN, SOC, and AP.

## Figures and Tables

**Table 1:** Soil physical and chemical properties of different land use types.

Soil Sample	TN (g/kg)	AN (mg/kg)	TP (g/kg)	AP (mg/kg)	TK (g/kg)	AK (mg/kg)	SOC (g/kg)
NF	1.672 ± 0.159 b	85.167 ± 5.09 b	0.264 ± 0.02 a	5.32 ± 0.67 b	6.25 ± 0.28 b	61.80 ± 4.32 a	71.47 ± 7.11 b
AF	4.166 ± 0.948 a	156.333 ± 21.61 a	0.479 ± 0.06 b	5.24 ± 0.85 b	9.76 ± 0.62 a	64.08 ± 20.46 a	64.23 ± 4.65 a
FL	3.155 ± 0.464 ab	135.333 ± 9.33 a	0.586 ± 0.32 b	30.76 ± 3.72 a	9.31 ± 0.334 a	86.42 ± 18.04 a	43.64 ± 3.77 a

Note: The data are means ± standard error (SE), different letters indicate significant levels (*p* < 0.05). a and b stand for Duncan’s multiple-range test. TN (total nitrogen); AN (immediate nitrogen); TP (total phosphorus); AP (immediate phosphorus); TK (total potassium); AK (immediate potassium); SOC (organic carbon); NF (natural forest); AF (artificial forests); FL (farmland).

**Table 2:** α Diversity of soil bacteria under different land uses.

Land Use Type	Chao1	Shannon	Simpson	Coverage
NF	4343.4 ± 14.939 a	10.2 ± 0.108 a	0.027 ± 0.001 b	0.974 ± 0.005 a
AF	3775.5 ± 159.176 b	10.41 ± 0.198 a	0.557 ± 0.011 a	0.974 ± 0.002 a
FL	4475.6 ± 116.811 a	9.6 ± 0.3555 b	0.193 ± 0.003 b	0.973 ± 0.003 a

Note: The data are means ± standard error (SE), different letters indicate significant levels (*p* < 0.05). a and b stand for Duncan’s multiple-range test.

**Table 3:** Spearman correlation analysis between soil physicochemical properties and microorganisms.

Phylum	Physical and Chemical Properties of the Soil						
	AK	AN	TN	TP	TK	AP	SOC
Myxococcota	0.2333	−0.569	−0.4833	−0.2333	−0.6667 *	0.35	−0.5167
Acidobacteriota	−0.2	0.2343	0.2333	−0.05	0.3833	−0.5333	0.4
Actinobacteriota	0.5	0.159	0.1333	0.45	−0.1333	0.6833 *	−0.1
Chloroflexi	0.4667	−0.1255	−0.0833	0.2333	−0.4	0.5167	−0.1
Bacteroidota	−0.3667	−0.0084	−0.15	−0.5333	−0.5833	−0.2833	−0.1353
Gemmatinnonadota	0.1333	−0.7029 *	−0.6833 *	−0.333	−0.8333 **	0.1167	−0.75 *
Planctomycetota	0.0833	0.3264	0.3533	−0.0333	0.35	−0.3833	0.5
Proteobacteria	−0.05	0.4268	0.3333	0.25	−0.0167	0.2667	0.3667
Verrucomicrobiota	0.1833	0.0418	0.0667	−0.0167	0.2167	0.0167	−0.1167
Chao1	0.35	−0.0335	−0.05	0.2833	−0.3833	0.4833	−0.3167
Shannon	0.4333	−0.1506	−0.1333	0.25	−0.4167	0.5	−0.4

Note: AK, AN, TN, TP, TK, AP, AK, AN, TN, TP, TK, AP, and SOC represent quick-acting potassium, quick-acting nitrogen, total nitrogen, total phosphorus, total potassium, quick-acting phosphorus, and organic carbon, respectively; * *p* < 0.05; ** *p* < 0.01. The bold indicates a significant correlation between soil physicochemical properties and soil bacterial phyla.

## Data Availability

The data that support the findings of this study are available on request from the corresponding author.

## Appendix A

**Table A1:** Summary of the high-throughput sequencing data.

Land Use Types	Sequences	Average Length	Coverage	OTU
NF	110,686	263.3	0.9775	5630
AF	109,092	263.2	0.9714	5102
FL	114,581	263.6	0.9776	5821

**Table A2:** ANOVA of dominant bacterial abundance at the phylum level (relative abundance > 1%).

Phylum	NF	AF	FL
Myxococcota	0.034 ± 0.003 a	0.022 ± 0.005 b	0.031 ± 0.006 ab
Acidobacteriota	0.175 ± 0.171 b	0.312 ± 0.06 a	0.146 ± 0.014 b
Actinobacteriota	0.14 ± 0.015 ab	0.113 ± 0.023 b	0.187 ± 0.008 a
Chloroflexi	0.073 ± 0.005 a	0.038 ± 0.003 b	0.086 ± 0.007 a
Bacteroidota	0.068 ± 0.01 a	0.061 ± 0.01 a	0.047 ± 0.002 a
Gemmatimonadota	0.077 ± 0.01 a	0.024 ± 0.002 b	0.063 ± 0.002 a
Planctomycetota	0.048 ± 0.002 ab	0.058 ± 0.004 b	0.037 ± 0.008 a
Proteobacteria	0.3 ± 0.002 a	0.315 ± 0.024 a	0.318 ± 0.019 a
Verrucomicrobiota	0.018 ± 0.004 a	0.015 ± 0.003 a	0.016 ± 0.005 a

Note: The data are means ± standard error (SE), different letters indicate significant levels (*p* < 0.05). a and b stand for Duncan’s multiple-range test.

**Table A3:** Summary of data on dominant phyla and diversity in different regions.

Different Regions	Dominant Soil Bacterial Phylum (Top 4 Abundance)				α Diversity
The results of this study Natural forests	Proteobacteria	Acidobacteriota	Actinobacteriota	Chloroflexi	Agricultural land had the highest Chao1 index, while natural forests had the lowest values. In addition, the Shannon diversity index was highest in natural forests and lowest in agricultural lands.
Artificial forests	Proteobacteria	Acidobacteriota	Actinobacteriota	Chloroflexi	
Farmland	Proteobacteria	Acidobacteriota	Actinobacteriota	Chloroflexi	
Sichuan [23] Sandy wetland	Proteobacteria	Actinobacteriota	Acidobacteriota	Chloroflexi	Chao1 index showed muddy wetlands > sandy wetlands > natural forest land > farmland
Farmland	Proteobacteria	Actinobacteriota	Acidobacteriota	Chloroflexi	
mud wetland	Proteobacteria	Actinobacteriota	Acidobacteriota	Chloroflexi	
natural forest land	Proteobacteria	Actinobacteriota	Acidobacteriota	Chloroflexi	
Guizhou [21] bare soil	Proteobacteria	Acidobacteriota	Chloroflexi	Actinobacteriota	The Chao1 and α diversity of soil bacteria in grassland are both at high levels and significantly higher than those in other land use types. and significantly higher than other land use types. The abundance and alpha diversity of soil bacteria in grassland are both at high levels and significantly higher than those in other land use types. and significantly higher than other land use types.
Farmland	Proteobacteria	Acidobacteriota	Chloroflexi	Actinobacteriota	
Grassland	Proteobacteria	Acidobacteriota	Chloroflexi	Actinobacteriota	
Shurb	Proteobacteria	Acidobacteriota	Chloroflexi	Actinobacteriota	
GuangXi [20] Farmland	Actinobacteriota	Proteobacteria	Acidobacteriota	Chloroflexi	No significant differences in the richness and diversity indices were found among the three plots. Chao, Simpson, and Shannon diversity indices were the highest in farmland, followed by grassland and plantations.
Grass land	Actinobacteriota	Proteobacteria	Acidobacteriota	Chloroflexi	
Artificial forest	Actinobacteriota	Proteobacteria	Acidobacteriota	Chloroflexi	
